# Role of Sex on the Genetic Susceptibility to Childhood Asthma in Latinos and African Americans

**DOI:** 10.3390/jpm11111140

**Published:** 2021-11-03

**Authors:** Antonio Espuela-Ortiz, Esther Herrera-Luis, Fabián Lorenzo-Díaz, Donglei Hu, Celeste Eng, Jesús Villar, Jose R. Rodriguez-Santana, Esteban G. Burchard, María Pino-Yanes

**Affiliations:** 1 Genomics and Health Group, Department of Biochemistry, Microbiology, Cell Biology and Genetics, Universidad de La Laguna, 38200 San Cristóbal de La Laguna, Spain; aespuela@ull.edu.es (A.E.-O.); estherhrl@outlook.com (E.H.-L.); florenzo@ull.edu.es (F.L.-D.); 2 Instituto Universitario de Enfermedades Tropicales y Salud Pública de Canarias (IUETSPC), Universidad de La Laguna, 38200 San Cristóbal de La Laguna, Spain; 3 Department of Medicine, University of California San Francisco, San Francisco, CA 94158, USA; donglei.hu@ucsf.edu (D.H.); Celeste.Eng@ucsf.edu (C.E.); Esteban.Burchard@ucsf.edu (E.G.B.); 4 CIBER de Enfermedades Respiratorias, Instituto de Salud Carlos III, 28029 Madrid, Spain; jesus.villar54@gmail.com; 5 Multidisciplinary Organ Dysfunction Evaluation Research Network (MODERN), Research Unit, Hospital Universitario Dr. Negrín, 35019 Las Palmas de Gran Canaria, Spain; 6 Centro de Neumología Pediátrica, San Juan 00917, Puerto Rico; jr@pedasthma.com; 7 Department of Bioengineering and Therapeutic Sciences, University of California San Francisco, San Francisco, CA 94158, USA; 8 Instituto de Tecnologías Biomédicas (ITB), Universidad de La Laguna, 38200 San Cristóbal de La Laguna, Spain

**Keywords:** asthma, sex interaction, sex-stratified, minority population, Hispanic, GWAS

## Abstract

Asthma is a respiratory disease whose prevalence changes throughout the lifespan and differs by sex, being more prevalent in males during childhood and in females after puberty. In this study, we assessed the influence of sex on the genetic susceptibility to childhood asthma in admixed populations. Sex-interaction and sex-stratified genome-wide association studies (GWAS) were performed in 4291 Latinos and 1730 African Americans separately, and results were meta-analyzed. Genome-wide (*p* ≤ 9.35 × 10^−8^) and suggestive (*p* ≤ 1.87 × 10^−6^) population-specific significance thresholds were calculated based on 1000 Genomes Project data. Additionally, protein quantitative trait locus (pQTL) information was gathered for the suggestively associated variants, and enrichment analyses of the proteins identified were carried out. Four independent loci showed interaction with sex at a suggestive level. The stratified GWAS highlighted the 17q12-21 asthma locus as a contributor to asthma susceptibility in both sexes but reached genome-wide significance only in females (*p*-females < 9.2 × 10^−8^; *p*-males < 1.25 × 10^−2^). Conversely, genetic variants upstream of ligand-dependent nuclear receptor corepressor-like gene (*LCORL*), previously involved in height determination and spermatogenesis, were associated with asthma only in males (minimum *p* = 5.31 × 10^−8^ for rs4593128). Enrichment analyses revealed an overrepresentation of processes related to the immune system and highlighted differences between sexes. In conclusion, we identified sex-specific polymorphisms that could contribute to the differences in the prevalence of childhood asthma between males and females.

## 1. Introduction

The divergence between sexes, often called sexual dimorphism, ranges in the animal kingdom from physical/external characteristics to physiology, and the human species is no exception. The effect of biological sex on a phenotype can be classified as sex-specific, when it only affects one sex, differential when it has a more pronounced effect in one sex, or temporal, when the effect manifests differently across time in each sex [1]. Previous research on sexual dimorphism in a wide range of processes such as coagulation, innate immunity, synthesis of hormones, or androgen sensibility, highlights that biological sex influences gene expression [1]. Furthermore, biological sex not only affects gonadal cells and reproduction through the effect exerted by sexual hormones, but it also affects non-sexual processes in non-reproductive cells through the genetic information encoded in the pseudo-autosomal region of the sex chromosomes. Additionally, autosomal variants also influence sexual dimorphism [2]. Nevertheless, not only physiological processes but disease-related traits show sex-specific differences [3,4]. In fact, sexual dimorphism has been reported to influence the prevalence, severity, or heritability of several diseases [3,4].

One human disease affected by sexual dimorphism is asthma [2], a respiratory condition whose symptoms are dyspnea, shortness of breath, cough, and chest tightness, due to the airways narrowing [5]. Risk factors for developing this disease are both extrinsic and intrinsic. The most important extrinsic factors include the characteristics of the surrounding environment, such as geographical location, climatic features, and the exposure to pollutants, tobacco smoke, or microbes [6]. On the other hand, among the intrinsic factors, some of the most studied are genetic variants, genetic ancestry, obesity, family history, allergic comorbidities, age, and biological sex [6,7,8,9,10,11].

Regarding the interaction of age, biological sex, and asthma prevalence, different trends coexist. During childhood, asthma is more prevalent among males, while after puberty its prevalence is higher in females [12,13]. Moreover, data from the Centers for Disease Control and Prevention (CDC) of the United States of America confirm the sex differences in asthma prevalence and highlight that some of the ethnic minority groups of the United States of America (e.g., Puerto Ricans and African Americans) show the highest prevalence of asthma regardless of sex and age group [14]. Additionally, some studies have highlighted differences in asthma prevalence occurring just after puberty and menopause, suggesting an important role of sex hormones and other physiological changes occurring during these periods [15,16], which could act through airway inflammation modulation [15].

There have been few epigenomic, transcriptomic, and genetic studies on sex interaction with asthma susceptibility published to date. In the Isle of Wight Birth Cohort, a sex-specific methylation CpG site was associated with asthma in infants and young adults [17]. Furthermore, the existence of sex-specific differentially expressed genes between asthma patients and controls in several tissues was reported by Gautam et al. [18]. Specifically, some asthma-related genes exhibit differences in expression magnitude and/or opposite expression patterns between sexes [18]. In the context of genetic studies, there is also previous work that has tackled this topic. In a candidate-gene association study in individuals of European descent, genotype-by-sex interaction at interferon-gamma (*IFNG*) was shown to affect the risk for childhood asthma [19]. Moreover, Mersha et al. flagged the interaction of sex and genetic variants located in 20 asthma-related genes on childhood asthma susceptibility in Europeans, highlighting sex-specific associations that were unidentifiable when both sexes were analyzed jointly [2]. Additionally, Myers et al. conducted genome-wide analyses in multiple populations of the EVE consortium (European American, African American/African Caribbean, and Latino) comparing cases of one sex against individuals without asthma and individuals with asthma of the other sex (pooled controls) [20]. Each ancestry group was studied separately, as well as meta-analyzed, performing sex-combined and sex-stratified analyses. Although no genome-wide significant variants were reported for any of the ancestry groups studied, six regions of sex-specific asthma were identified at a suggestive significance level (*p*-value < 1 × 10^−6^), and two of them correlated with nearby gene expression [20]. Finally, Gauderman et al. recently proposed a unified model that considers the genotype of each genetic variant as the outcome and allows reporting the effect of the phenotype on genotype, the effect of an environmental variable on genotype, and the interaction effect of genotype and the environmental variable [21]. Applying this model to a cross-sectional childhood cohort, the authors detected three genomic regions (9p13.3, 9p24.3, and 5q13.32) with different patterns of association with asthma [21].

All the above highlights that sex has been scarcely explored in genomic studies despite being a crucial factor. In fact, in most genetic studies performed to date, sex has only been considered as a confounder factor. Since precision medicine of asthma aims to offer an individualized diagnosis, prognosis, and treatment, understanding the role of the biological sex in this disease could aid in meeting those goals. This study hypothesized that sex-driven differences may impact asthma susceptibility and that a genomic approach could reveal additional genetic variants involved in asthma pathogenesis. We aimed to identify genetic variants whose effects on childhood asthma susceptibility are influenced by biological sex. For that, we performed sex-interaction and sex-stratified genome-wide association studies (GWAS) in two populations with a high asthma burden, African Americans and Latinos.

## 2. Results

### 2.1. Study Populations

The genetic role of sex on asthma susceptibility was assessed in the Genes-environments & Admixture in Latino Americans (GALA II) and the Study of African Americans, Asthma, Genes, and Environments (SAGE) studies [22]. The clinical and demographic characteristics of the 6021 individuals (4291 Latinos and 1730 African Americans) analyzed are depicted in Table 1. The proportion of males was higher among cases compared to controls in both studies, concordant with the age range of the recruited population (8–22 years old). Age differed significantly between cases and controls, and males were younger than females (*p* = 2.1 × 10^−20^). Overall, GALA II participants were younger than individuals from SAGE (*p* = 6.6 × 10^−35^). Controls were significantly taller than cases in all the comparisons performed. Latino individuals had lesser African ancestry compared to African American individuals regardless of the analysis. Additionally, in the Latino group, Native American ancestry was significantly higher in controls than in cases, except in the male-only analyses.

### 2.2. Significance Threshold Estimation

The most used genome-wide threshold to declare statistical significance for GWAS (*p* ≤ 5 × 10^−8^) has been defined based on European populations [23]. Since the populations included in this work were from a different genetic background (i.e., African Americans and Latinos), new population-specific thresholds for declaring statistical significance were calculated based on Kanai et al. [24], as detailed in the Methods section. After selecting those variants with MAF ≥ 1% (14,119,721) and in low linkage disequilibrium (LD), a total of 534,667 independent variants were detected. Based on this, the suggestive threshold was set to 1.9 × 10^−6^, and the genome-wide genome threshold was defined as 9.4 × 10^−8^ for transethnic meta-analyses of African American and Latino populations.

### 2.3. Interaction GWAS

**Figure 1:**
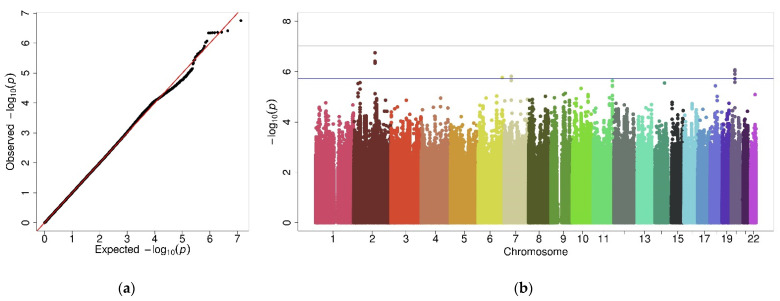
Graphic representation of the results from interaction GWAS. (**a**) Quantile-Quantile (Q-Q) plot showing the distribution of observed vs. expected *p*-values. (**b**) Manhattan plot showing the genetic variants association results. The *X*-axis shows the chromosomal position for each variant and the *Y*-axis shows a transformation of the association *p*-value (−log_10_ [*p*-value]). The horizontal grey line shows the genome-wide significance threshold (*p* = 9.4 × 10^−8^), and the blue line shows the suggestive significance threshold (*p* = 1.9 × 10^−6^).

No evidence of genomic inflation was observed based on the Quantile-Quantile (Q-Q) plot for the 6,680,224 variants analyzed (Figure 1a) or the lambda value (λ = 0.97). Even though no genome-wide significant variants were detected, 13 variants showed suggestive interaction terms (9.35 × 10^−8^ ≤ *p* ≤ 1.87 × 10^−6^) (Figure 1b). For these variants, the alternative allele had opposite effects in each sex (protective effect in females and risk in males, except for variants rs6114734 and rs61548213 in which the effects were inverted) (Table S1). Independency among variants in each analysis performed was assessed with the PLINK 1.9 clumping method (distance = 1 Mb and LD *r*^2^ = 0.2). Table 2 summarizes the results of the four independent variants with a suggestive sex interaction effect, located at 2q22.1, 6q25.3, 7p12.3, and 20p11.21.

### 2.4. Stratified GWAS in Females

**Figure 2:**
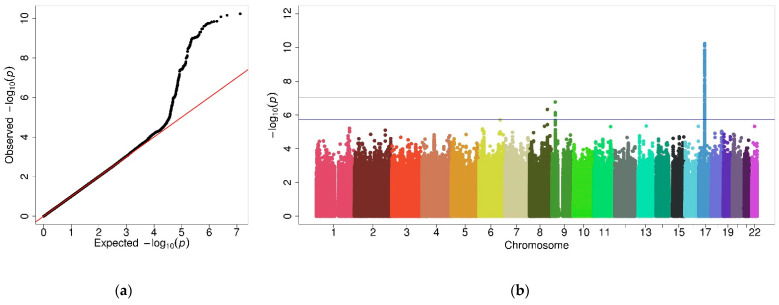
Graphic illustration of the results from female GWAS. (**a**) Q-Q plot showing the distribution of observed *p*-values vs. the distribution of expected *p*-values. (**b**) Manhattan plot showing the association results of genetic variants. The *X*-axis represents the chromosomal position for each variant and the *Y*-axis shows a transformation of the association *p*-value (−log_10_ [*p*-value]). The horizontal grey line indicates the genome-wide significance threshold (*p* = 9.4 × 10^−8^), and the horizontal blue line indicates the suggestive threshold (*p* = 1.9 × 10^−6^).

A total of 6,680,224 variants were analyzed in 3075 females. Eighty-three variants in high LD at chromosome 17 exceeded the genome-wide significance threshold (*p*-value < 9.4 × 10^−8^) (Figure 2a and Table S2). The Q-Q plot showed deviation from the expected distribution of *p*-values (Figure 2b), despite having no sign of genomic inflation (λ = 0.97). The examination of the Q-Q plot excluding variants on chromosome 17 (Figure S1) suggested that the deviation was caused by the association signal at 17q12-21. Independent variants within each genomic region are depicted in Table 3.

Variants in 17q12-21 were investigated in males to assess their sex-specificity, and all of them were nominally associated in males but to a lesser degree (0.01 ≥ *p* ≥ 8 × 10^−5^) and their effects were consistent with the ones found in females (Table S2).

### 2.5. Stratified GWAS in Males

**Figure 3:**
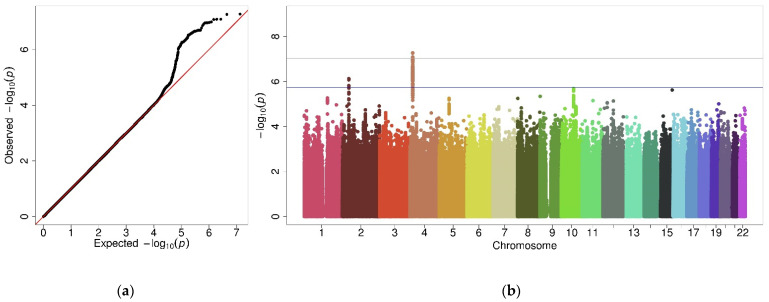
Graphic representation of results from male GWAS. (**a**) Q-Q plot of *p*-values and (**b**) Manhattan plot showing the association of genetic variants. The *X*-axis shows the chromosomal position for each variant and the *Y*-axis shows a transformation of significance (−log_10_[*p*-value]). The horizontal grey line shows the genome-wide significance threshold (*p*-value = 9.4 × 10^−8^) whereas the suggestive significance threshold (*p*-value = 1.9 × 10^−6^) is represented by the blue line.

A total of 6,680,224 variants were analyzed in 2946 individuals. Four variants near lactate dehydrogenase A pseudogene 3 (*LDHAP3*) on 2p21 and 90 variants upstream of the ligand dependent nuclear receptor corepressor-like (*LCORL*) gene on region 4p15.31 were suggestively associated with asthma, five of them reaching the genome-wide significance (Figure 3 and Table S3). Of these 94 variants, two were identified as independent, one at *LDHAP3* and the other at the *LCORL* region (Table 3). The complete summary of the results is presented in Table S3.

The asthma-associated variants in 2p21 near *LDHAP3*, a locus previously associated with severe asthma exacerbations in Latinos from GALA II [25], had a predominant effect in males and were nominally significant in the interaction and female-only analyses (*p*-value < 0.05) (Table 3 and Table S3). However, variants upstream of *LCORL* at 4p15.31 had sex-specific involvement in male asthma susceptibility, being nominally significant in the interaction GWAS (*p*-value < 0.05), but not in females (*p*-value > 0.05) (Table 3 and Table S3).

Given that *LCORL* has been related to height measurements [26,27,28,29], sensitivity analyses were performed to assess the confounder effect of this variable on our results. The association between the genetic variants and asthma susceptibility was assessed by two regression models adjusted/unadjusted by height as a covariate, including only individuals with height measurements. After performing the analyses, the potential confounding effect of height was discarded, since results barely changed with the adjustment (association results for the top hit [rs4593128]: *p*_unadjusted_ = 5.63 × 10^−6^; *p*_heigh-adjusted_ = 5.03 × 10^−6^).

### 2.6. PQTL Analyses and Enrichment Analysis

The proteins regulated by the associated variants in each of the stratified analyses were subjected to enrichment analyses to assess their contribution to asthma susceptibility. The results obtained from the protein quantitative trait locus (pQTL) enrichment showed the implication of these variants via immune response regulation and response to several stimuli, as well as through sexual differentiation, and tissue development. Other terms related to apoptosis and cell cycle were also detected. When analyzing the pQTLs of the variants associated in females and males separately, even though some of the terms were common to both sexes, and the global categories were shared, there were terms involving different mechanisms that were sex-specific. As an example, terms related to response to reactive oxygen species/apoptosis, blood coagulation/platelet activation/wound healing, glucose transport, or viral cell cycle were female-specific, while interleukin production or T-cell mediated immune response were found only in males (Table S4).

### 2.7. Validation of Variants Previously Reported Having a Sex-Specific Role in Asthma or an Interaction Effect

An attempt to replicate previous sex-specific genetic associations was carried out. Results from Mersha et al. [2] reporting variants with *p* ≤ 0.05 in sex-combined or sex-stratified analyses, and an effect size (OR) lower than 0.69 or greater than 1.44, as estimated by the authors as the threshold to consider significant associations [2], were followed up for replication in our study. Among those variants, rs2243250 (*IL4*, *p* = 0.021) and rs2227562 (*PLAU*, *p* = 0.004) were nominally replicated in our interaction GWAS. Additionally, rs11102221 (*CHI3L2*, *p* = 0.037), rs12006123 (*RIG-I*, *p* = 0.022), and rs2227562 (*PLAU*, *p* = 0.028) were nominally replicated in females, and rs3806933 (*TSLP*, *p* = 0.037), rs2289277 (*TSLP*, *p* = 0.015), rs2243250 (*IL4*, *p* = 0.023), rs7445392 (*SPINK5*, *p* = 0.019), rs6892205 (*SPINK5*, *p* = 0.037), and rs1805011 (*IL4R*, *p* = 0.019) were nominally replicated in males. The complete replication results are summarized in Table S5.

Myers et al. [20] revealed six loci suggestively associated (*p* ≤ 10^−6^) with asthma susceptibility in admixed populations when stratifying by sex. Four SNPs from their results had MAF > 0.05 in our data and were tested for replication (Table S6). From those, the SNP rs4673659, located on the Erb-B2 receptor tyrosine kinase 4 (*ERBB4*) gene was nominally significant but with the effect in the opposite direction in the male-only results from our study. The other three variants were not associated in any of the analyses (*p*-value > 0.05).

## 3. Discussion

In this study, we assessed the role of sex in asthma susceptibility in two African-admixed populations (African Americans and Latinos) by means of two different genetic association analyses, interaction with sex, and sex-stratified approaches. Meta-analyses of both populations highlighted several genomic regions as potentially influenced by sex. Although this is not the first study focused on sex-interaction with asthma susceptibility, this is the first to report genome-wide significant variants for each sex. We uncovered a new locus specifically associated with asthma in males only, and our results supported the role of the genomic region 17q12-21 in both sexes. Additionally, we aimed to replicate variants previously highlighted by studies using a similar methodology and were able to nominally replicate the associations described in a candidate–gene association analysis. Lastly, network analyses of the proteins regulated by the associated variants showed that asthma may act through different mechanisms in each sex despite involving the same biological processes.

Interaction analyses revealed several variants at 2q22.1, 6q25.3, 7p12.3, and 20p11.21 that had a suggestive interaction with sex, potentially influencing asthma susceptibility by modulating expression or methylation of nearby genes. Variants from 2q22.1 have been described as a brain splicing quantitative trait locus (sQTL) of the Histamine N-methyltransferase gene (*HNMT*). Moreover, the intronic variant rs73021932 located in Synaptojanin 2 gene (*SYNJ2*) in 6q25.3 is known to act as blood methylation quantitative trait locus (meQTL). Furthermore, variants rs12539647 and rs6036804 had also been described as blood expression quantitative trait locus (eQTLs) of *IGFBP3* and cystatin F (*CST7*), respectively. Among these genes, most have been associated with immune response or asthma, such as *HNMT*, *SYNJ2*, or *CST7*. *HNMT* has been linked to allergic asthma through its role in histamine levels regulation in different tissues, such as respiratory-related ones [30]. *SYNJ2* is a gene involved in clathrin-dependent endocytosis and it is implicated in receptor internalization as well as in cell migration [31], processes related to antigen processing. *SYNJ2* has several splice variants, and some of them are expressed predominantly in the testis and the brain [31], which could be the reason why it is detected in interaction analyses. Lastly, *CST7* is a gene that participates in eosinophil survival in the lungs, which potentially impacts asthma pathogenesis [32].

The well-known asthma locus 17q12-21 in our study also showed an association with susceptibility to asthma, in both sexes. The link between asthma and this locus was revealed in the first GWAS of asthma [33] and has been the most replicated region throughout the years in different asthma phenotypes and asthma-related traits [34]. Based on distinctive LD patterns, which are not completely shared among populations, this locus is divided into proximal, core, and distal regions [35]. The variants associated in the current study encompassed core genes of the 17q12-21 locus: growth factor receptor-bound protein 7 (*GRB7), IKAROS* family zinc finger 3 (*IKZF3*), zona pellucida binding protein 2 (*ZPBP2*), gasdermin B (*GSDMB*), *ORMDL* sphingolipid biosynthesis regulator 3 (*ORMDL3*), and *AC090844.2* [35]. Those variants have been reported to act as eQTL and meQTL of several genes in this region in different tissues [36,37], supporting the involvement of this locus in asthma susceptibility through changes in transcription and methylation [35,38].

Variants upstream *LCORL* gene had a sex-specific involvement in male asthma susceptibility. The asthma-associated variants at that locus (4p15.31) have been shown to act as eQTL of *LCORL* in kidney tissue cells, and the quinoid dihydropteridine reductase (*QDPR*) gene in blood. Additionally, they regulate methylation levels of several CpG sites (cg23562514, cg01886556, cg03590257, cg08925142, and cg13111374) which are annotated to *LCORL*, *FAM184B*, and *DDB1* and *CUL4* associated factor 16 (*DCAF16*) genes in blood monocytes and neutrophils. *LCORL* is a gene involved in spermatogenesis, but little is known about its function. Genetic variants in *LCORL* have been associated with familiar squamous cell carcinoma [39] and with biometric variables, such as birth weight and height in different stages of life (fetal growth, childhood, and adulthood) [26,27,28,29]. Given these associations, sensitivity analyses were performed to assess the confounder effect of height on our results, but this variable did not explain the association with asthma. Furthermore, variant rs4593128, the top hit association in this locus, has been previously described as a protein quantitative trait locus (pQTL) of 35 proteins (Table S7). Among the proteins whose levels are regulated by the variant rs4593128, some genes previously associated with asthma (i.e., a disintegrin and metalloproteinase with thrombospondin motifs 1 [*ADAMTS1*] and thymic stromal lymphopoietin [*TSLP*]) [40,41], and some genes related to the immune system (anaphase-promoting complex subunit 7 [*ANAPC7*], annexin A1 [*ANXA1*], collectin-12 [*CL-12*], interferon lambda-1 [*IFNL1*], and interleukin-18 receptor 1 [*IL18R1*]) [42] were included.

With regards to the sex-stratified enrichment analyses of suggestive variants, in both sexes, most of the terms were related to the regulation of the immune response as expected since asthma has an important immunological component. In fact, the enrichment observed for terms related to apoptosis and cell cycle could be related to the role of these processes in the training that some types of immune cells need to undergo [43]. Interestingly, despite sharing terms between sexes, differences were also found, possibly pointing out differences in the pathological molecular mechanisms involved in each sex.

Despite sex hormones have been pointed as the main contributor to the differences in asthma between sexes [44], no genetic variants in sex hormones’ genes or in genes from related pathways were detected in females nor in males in this study. However, proteins being regulated by genetic variants from the female GWAS revealed a significant enrichment in a term related to the estrogen pathway.

This study has some limitations that need to be acknowledged. First, despite the use of new population-specific significance thresholds which enabled us to detect several loci associated with asthma susceptibility in Latino and African Americans, joint estimation based on three different populations together may not be the optimal procedure. However, this approach represents a step forward, since previous estimations were calculated from European populations that have more distinctive LD patterns. Second, the study was powered at 80% to detect associations with MAF and relative risk (RR) > 1.55 for the interaction analysis, and this power was smaller in the stratified GWAS for the same allele frequency and RR (females: 20.8% and males: 17.8%). In fact, for the stratified analyses, 80% power was achieved for RR > 1.8 in females and RR > 1.8 in males for variants with MAF ≥ 0.05 [45]. For this reason, despite rare variants could have a role in the sex-differences in asthma susceptibility, they were not analyzed in our study. Third, we analyzed asthma as a unique syndrome without stratifying by asthma phenotypes which could potentially veil some associations that could be subphenotype-specific. Finally, the functional data analyzed was queried from quantitative trait locus (QTL) databases where both sexes have been analyzed jointly, which could confound possible differences in the influence of genetic variants in expression or methylation since the existence of sex-specific eQTLs has been reported [46].

On the other hand, the strengths of this study include the analysis of minority populations, largely understudied despite being amongst the most affected by asthma worldwide, and the application of different types of analysis that allowed us to tackle sex influence on asthma susceptibility from different perspectives. These results show that the contribution of sex in asthma pathogenesis needs further research for its understanding and future use in the clinic.

In conclusion, we revealed a new male-specific locus associated with asthma at 4p15.31 that regulates protein levels of several immune-related proteins and proteins involved in asthma pathogenesis, validated the role of 17q12-21 in both sexes, and highlighted the interaction of sex with four loci. These results suggest that despite sharing several mechanisms, asthma pathogenesis may also act through different pathways in each sex.

## 4. Materials and Methods

### 4.1. Study Sample

GALA II and SAGE are two clinic-based case-control studies focused on untangling the genetic and environmental basis of childhood-onset asthma disease in minority ethnic groups of the United States of America [22]. At the time of enrolment, all participants were between 8 and 22 years old. All participants/parents gave written informed consent/assent to participate in the study. The University of California, San Francisco (UCSF) and each study site’s institutional review board (IRB) approved the SAGE II/GALA II protocols (SAGE II UCSF-IRB No. 10-02877; GALA II UCSF-IRB No. 10-00889). Inclusion criteria were self-identification as Latino or African American for GALA II or SAGE, respectively. Cases were subjects with a physician diagnosis of asthma and manifestations of symptoms within the two years before recruitment, while controls were selected based on the absence of a medical history of asthma, allergic diseases, or other respiratory affections. Recruitment was performed in different centers throughout mainland United States and Puerto Rico for GALA II subjects, while SAGE participants were recruited in the San Francisco Bay Area. For the current analyses, a subset of 6021 individuals (4291 Latinos and 1730 African Americans) was selected based on genotype and demographic data availability.

### 4.2. Genotyping, Imputation of Genetic Variants and Ancestry Assessment

Genotyping was carried out with the Axiom LAT1 array (817,810 genetic variants) and the Axiom LAT1 Array Plus HLA (812,715 SNPs) (Affymetrix), as described elsewhere [25], and joined quality control was carried out for the subset of 803,225 variants resulting from the intersection of the two arrays. Quality control main steps included the exclusion of individuals with: low call rate (<97%), a discrepancy between reported gender and biological sex, relatedness with other subjects (Pi-hat > 0.3), and evidence of being outliers for any of the three first principal components of the genotype matrix (>six standard deviations) estimated with PLINK 1.9 [47]. Additionally, genetic variants were excluded if they had: low call rate (<95%) or deviated from Hardy–Weinberg equilibrium in the control group (*p*-value ≤ 10^−6^), as described elsewhere [25].

To expand the coverage along the genome, imputation of genetic variants was performed on the Michigan Imputation Server [48] using the 1000 Genomes Project Phase 3 version 5 [49] as a reference panel, analyzing GALA II and SAGE separately. Specifically, haplotype reconstruction was performed with SHAPEIT v2.r790 [50] and imputation was carried out with Minimac3 [48]. After imputation SNPs were filtered based on imputation quality (R^2^ ≥ 0.3).

Genetic ancestry was estimated with ADMIXTURE [51]. Reference haplotypes included European (CEU) and African (YRI) populations from HapMap Project Phase III for African Americans, as well as 71 Native Americans (NAM) for Latinos [52].

### 4.3. Significance Threshold Estimation

Currently, the statistical significance threshold (5 × 10^−8^) is set based on an estimation of a million independent common variants drawn from the genome of European individuals [23]. Since the analyzed populations have a different genetic background, which differs in terms of LD, we recalculated that threshold in order to reduce the number of false-negative associations. Population-specific thresholds were estimated following the procedures described in Kanai et al. [24]. Briefly, available data from 1000 Genomes Project (1KGP) Phase 3 data version 5 were downloaded from http://www.1000genomes.org/ (accessed 1 March 2020) and African American (ASW, *n* = 61), Mexican (MXL, *n* = 64) and Puerto Rican (PUR, *n* = 104) unrelated individuals were selected since these populations are the ones that best represent the populations in our dataset. Parameters proposed by Kanai et al. were modified to fit a different scenario (MAF ≥ 0.01; LD R^2^ = 0.2; sliding window size = 40 kb; window step size = 4 kb).

The thresholds to declare genome-wide and suggestive significance were estimated adopting a Bonferroni correction approach for multiple comparisons, defining them as the quotient between the number of false positives expected per individual analysis and the number of independent variants detected. Thresholds for the combined population that englobes ASW, MXL, and PUR were calculated considering variants after deduplication of multiallelic variants and those that shared chromosomic position. For the suggestive significance level, the number of false positives (α) was considered to be 1 whereas, for the genome-wide significance, 0.05 was considered [53].

### 4.4. Statistical Analysis

The relation between sex and asthma and common genetic variants (MAF ≥ 0.05) was assessed using two different approaches. First, logistic regression models including a *SNP*-by-sex interaction term were used. Second, sex-stratified analyses were carried out. The use of the first approach (interaction) provides insights into variants that have a differential effect between sexes (that is, positive effect in one sex and negative in the other, or no effect in one sex but positive/negative effect on the other). The second approach (sex-stratified) allows the detection of variants that affect disease susceptibility only in one sex. Both approaches were performed using PLINK 2.0 software [47] on allelic dosages for each variant (range from 0 to 2), considering the genotype effect as additive. The absence/presence of asthma was considered as the dependent binary variable, and sex was coded as 1 for males and 2 for females in the logistic regression models adjusted by age, sex (only in interaction GWAS), and the top first two PCs of the genotype matrix according to the following models:

Interaction: Asthma ~ SNP + (SNP × Sex) + Sex + Age + PC1 + PC2(1)

Sex-Stratified: Asthma ~ SNP + Age + PC1 + PC2(2)

PC: Principal component of the genotype matrix.

Each study was analyzed separately and then meta-analyzed using the random-effect model (RE2) implemented in METASOFT [54]. Independent variants for each of the analyses were assessed with the PLINK 1.9 clumping method (distance = 1 Mb and LD *r*^2^ = 0.2). SNP nexus web interface was used to obtain the reference identifier for each variant (rsID), and annotation to the closest gene was performed by the Variant Annotation Integrator (UCSC) using the Comprehensive Gene Annotation Set from GENCODE Version 37lift37 (Ensemble 103) as a reference. Statistical power for each analysis was estimated with the Genetic Association Study (GAS) Power calculator [45].

Sensitivity analyses were conducted for the variants in locus 4p15.31 to assess the effect of height (centimeters) since height measurements were not initially included in the regression models. For that, two regression models adjusted/unadjusted by the confounder, including only individuals with complete height data were compared.

### 4.5. In Silico Analysis

Different online databases were queried to assess the potential functional effect of the associated variants. Briefly, the role of the significant/suggestive variants in the regulation of the expression (eQTL) or methylation (meQTL) of close genes was assessed using the Genotype-Tissue Expression (GTEx) v8 database [36], QTLbase [55], eQTLGen consortium data [56], and Phenoscanner v2 [57]. Moreover, Phenoscanner database v2 [57] was also used to query pQTL information for the associated variants in stratified analyses separately (catalogue = pQTL, *p* = 0.01, no proxies, build = 37). Enrichment analysis of the list of proteins regulated by those variants was performed within the STRING version 11.0 online interface [58] and all the reported terms with a strength value greater than zero (indicating a larger proportion of observed genes than expected within a term) and a false discovery rate (FDR) < 0.01 were considered. All possible enrichment sets were explored for each sex separately, and terms were manually classified attending to their nature.

## Figures and Tables

**Table 1:** Characteristics of subjects included in the analyses.

	GALA II (*n* = 4291)			SAGE (*n* = 1730)		
	Cases (*n* = 2263)	Controls (*n* = 2028)	*p*-Value	Cases (*n* = 1108)	Controls (*n* = 622)	*p*-Value
Characteristic	Overall (*n* = 6021)					
Sex (% female)	1031 (45.6)	1137 (56.1)	**7.8 × 10^−12^**	549 (49.5)	358 (57.6)	**1.6 × 10^−3^**
Age (years)	12.7 ± 3.3	13.9 ± 3.6	**7.0 × 10^−28^**	14.0 ± 3.6	15.8 ± 3.8	**2.5 × 10^−20^**
Height (cm) ^a^	151 ± 14.0	155.5 ± 13.1	**3.4 × 10^−18^**	157.9 ± 14.6	162 ± 13.9	**1.2 × 10^−6^**
African ancestry (%)	16.2 ± 13.1	14.1 ± 12.0	**4.4 × 10^−8^**	78.9 ± 11.4	78.1 ± 11.4	0.06
European ancestry (%)	54.6 ± 18.8	52.0 ± 20.9	**1.4 × 10^−3^**	21.1 ± 11.4	21.9 ± 11.4	0.06
Native American ancestry (%)	29.2 ± 25.0	33.9 ± 27.8	**4.8 × 10^−6^**	NA ^b^	NA ^b^	NA ^b^
Characteristic	Female (*n* = 3075)					
Age (years)	13.3 ± 3.6	14.1 ± 3.6	**1.2 × 10^−8^**	14.4 ± 3.8	16.0 ± 3.8	**8.4 × 10^−10^**
Height (cm) ^a^	150.5 ± 11.9	153.2 ± 10.3	**7.9 × 10^−5^**	156.9 ± 11.1	159.7 ± 10.8	**4.2 × 10^−4^**
African ancestry (%)	16.5 ± 13.1	14.0 ± 12.1	**5.9 × 10^−7^**	78.5 ± 12.4	78.3 ± 11.0	0.08
European ancestry (%)	54.9 ± 18.7	51.4 ± 21.1	**9.9 × 10^−4^**	21.5 ± 12.4	21.7 ± 11.0	0.08
Native American ancestry (%)	28.6 ± 25.0	35.0 ± 28.0	**4.9 × 10^−6^**	NA ^b^	NA ^b^	NA ^b^
Characteristic	Male (*n* = 2946)					
Age (years)	12.3 ± 3.1	13.6 ± 3.6	**4.1 × 10^−18^**	13.5 ± 3.4	15.4 ± 3.9	**1.5 × 10^−10^**
Height (cm) ^a^	151.3 ± 15.6	158.5 ± 15.4	**6.8 × 10^−17^**	158.9 ± 17.4	165 ± 16.6	**1.1 × 10^−4^**
African ancestry (%)	16.0 ± 13.0	14.4 ± 12.0	**8.6 × 10^−3^**	79.2 ± 10.3	77.8 ± 12.0	0.33
European ancestry (%)	54.3 ± 18.8	52.7 ± 20.7	0.27	20.8 ± 10.3	22.2 ± 12.0	0.33
Native American ancestry (%)	29.7 ± 24.9	32.9 ± 27.4	0.06	NA ^b^	NA ^b^	NA ^b^

^a^ Height data was gathered for 3253 individuals from GALA II (579 female controls, 1010 female cases, 454 male controls, and 1210 male cases) and 1445 individuals from SAGE (200 female controls, 541 female cases, 158 male controls, and 546 male cases). ^b^ Estimates of Native American ancestry are not available (NA) for SAGE since only two ancestral components (African and European) were considered for this population. Continuous variables are presented as the mean ± standard deviation. Categorical variables are presented as the number of individuals and percentage in parenthesis. Chi-square test and Wilcoxon test were used for detecting statistical differences between cases and controls for categorical/continuous variables, respectively. Statistically significant differences are highlighted in bold (*p* ≤ 0.05).

**Table 2:** Summary of independent suggestive variants identified (*p*-value < 1.9 × 10^−6^) in the interaction GWAS and their association results in stratified analyses.

rsID	Chr:pos ^a^	A1/A2	Closest Gene	OR (95% CI) Interaction	*p*-Value Interaction	OR (95% CI) Female	*p*-Value Female	OR (95% CI) Male	*p*-Value Male
rs146406602	2:139218399	CA_7_/C ^b^	*AC114763.1*	0.56 (0.45–0.70)	1.76 × 10^−7^	0.76 (0.63–0.92)	6.09 × 10^−4^	1.39 (1.19–1.64)	6.42 × 10^−5^
rs73021932	6:158513756	T/C	*SYNJ2*	0.55 (0.29–1.08)	1.72 × 10^−6^	0.70 (0.51–0.98)	1.48 × 10^−5^	1.26 (0.91–1.73)	2.48 × 10^−2^
rs12539647	7:46170582	C/T	*AC023669.2*	0.70 (0.60–0.81)	1.53 × 10^−6^	0.82 (0.74–0.91)	3.10 × 10^−4^	1.18 (1.05–1.32)	6.01 × 10^−3^
rs6036804	20:24433071	C/A	*GAPDHP53*	0.63 (0.39–1.01)	8.49 × 10^−7^	0.86 (0.77–0.95)	5.72 × 10^−3^	1.36 (0.90–2.04)	2.43 × 10^−5^

^a^ Coordinates are referred to the GRCh37 reference genome. ^b^ CA_7_ refers to the insertion of up to 7 copies of nucleotide A. A1: effect allele; A2: non-effect allele; Chr: chromosome; CI: confidence interval; OR: odds ratio; pos: position; rsID: Reference SNP ID; SE: standard error.

**Table 3:** Summary of independent variants identified in the stratified analyses (*p* ≤ 1.9 × 10^−6^), and their association results in the interaction analyses.

Analysis	rsID	Chr:pos ^a^	A1/A2	Closest Gene	OR (95% CI) Female	*p*-Value Female	OR (95% CI) Male	*p*-Value Male	OR (95% CI) Interaction	*p*-Value Interaction
Female	rs72683967	8:117337397	G/A	*LINC00536*	0.66 (0.49–0.89)	**4.70 × 10^−7^**	1.05 (0.89–1.23)	6.18 × 10^−1^	0.62 (0.40–0.97)	**1.43 × 10^−4^**
	rs4400476	9:22862535	T/G	*AL391117.1*	0.78 (0.60–1.00)	**1.71 × 10^−7^**	1.05 (0.91–1.21)	5.54 × 10^−1^	0.70 (0.60–0.82)	**1.08 × 10^−5^**
	rs907092	17:37922259	A/G	*IKZF3*	0.69 (0.54–0.89)	**5.85 × 10^−11^**	0.83 (0.67–1.02)	**4.51 × 10^−4^**	0.82 (0.69–0.97)	**2.72 × 10^−2^**
Male	rs10207164	2:42031062	C/T	*LDHAP3*	1.15 (1.01–1.33)	**4.89 × 10^−2^**	1.45 (1.25–1.68)	**7.50 × 10^−7^**	0.79 (0.64–0.96)	**1.89 × 10^−2^**
	rs4593128	4:18221770	G/T	*LCORL*	0.92 (0.78–1.07)	2.95 × 10^−1^	0.64 (0.54–0.75)	**5.31 × 10^−8^**	1.36 (1.09–1.70)	**7.38 × 10^−3^**

^a^ Coordinates are referred to the GRCh37 reference genome. A1: effect allele; A2: non-effect allele; CI: confidence interval; Chr: chromosome; OR: odds ratio; p: *p*-value; pos: position; rsID: Reference SNP ID; SE: standard error. Nominally significant associations (*p*-value < 0.05) are highlighted in bold.

## Data Availability

The data presented in this study are available on request from the corresponding author.

## Supplementary Materials

The following are available online at https://www.mdpi.com/article/10.3390/jpm11111140/s1, Table S1. Summary of the suggestive variants identified in interaction analysis (*p* ≤ 1.9 × 10^−6^), and their association results among females and males, Table S2. Summary of suggestive variants identified among females (*p* ≤ 1.9 × 10^−6^), their association results among males, and in the interaction analyses, Table S3. Summary of the suggestive variants identified among males (*p* ≤ 1.9 × 10^−6^), their association results among females, and in the interaction analyses, Table S4. Enrichment analysis results for the genes regulated by significant/suggestive variants in females and males, Table S5. Replication of results from Mersha et al., Table S6. Replication of findings from Myers et al. for associated variants with asthma susceptibility, Table S7. List of proteins regulated by variant rs4593128 extracted from PhenoScanner, Figure S1: Q-Q plot of *p*-values after excluding variants in chromosome 17 to assess the source of genomic inflation observed in the whole GWAS.
