# Supplementary material for: Role of Sex on the Genetic Susceptibility to Childhood Asthma in Latinos and African Americans
Source: J Pers Med. 2021 Nov 3;11(11):1140. doi: 10.3390/jpm11111140 (PMC8625344; doi:10.3390/jpm11111140)
Supplement: Supplementary file 1 [file jpm-11-01140-s001.zip › jpm-1402833-Figure S1.pdf]

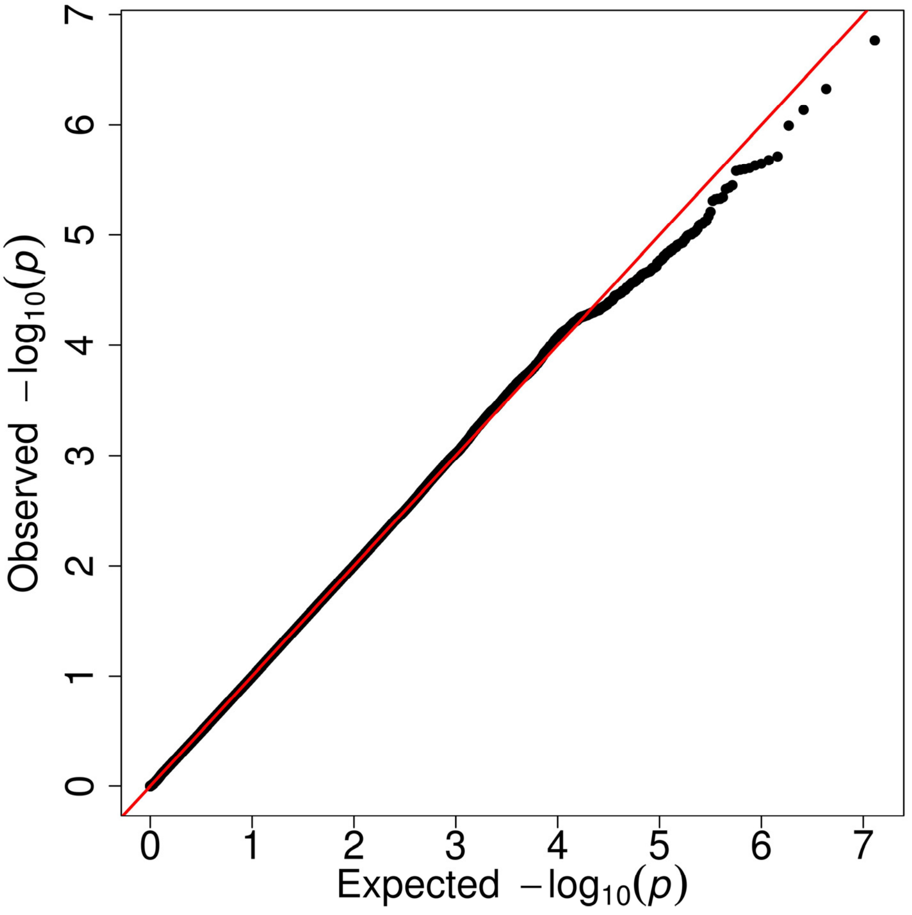

Figure S1. Q-Q plot of *p*-values after excluding variants in chromosome 17 to assess the source of genomic inflation observed in the whole GWAS.
